# Heat‐induced kidney disease: Understanding the impact

**DOI:** 10.1111/joim.20037

**Published:** 2024-11-28

**Authors:** Carl‐Gustaf Elinder

**Affiliations:** ^1^ Department of Clinical Sciences and Technology, Department of Renal Medicine Karolinska Institutet Stockholm Sweden

**Keywords:** agrochemicals, chronic kidney disease, epidemiology, heat stress, pathology

## Abstract

Research on Mesoamerican Nephropathy, chronic kidney disease of unknown cause and chronic kidney disease of nontraditional cause has been going on for more than 20 years. Thousands of manual workers, especially in agriculture, are affected. The disease has been reported in different countries and regions, not only from heat‐stressed sugarcane cutters in Central America but also from other occupational groups with strenuous work in hot environments. The cause of this disease is still debated. A multitude of causative factors have been suggested, including agrochemicals, water quality, infections, and heavy metals. The evidence that heat stress is the major cause of kidney disease is convincing, whereas the support for alternative causes is weak. Associations between exposure and kidney damage are strong, consistent, and specific, occur after acute and chronic exposure, display dose‐effect and dose–response relationships, are plausible, and coherent. Improving working conditions by providing hydration, rest, and shade to heat‐stress‐exposed workers is beneficial. Continued global warming will increase the number of people at risk for dangerous heat exposure and kidney disease.

## Graphical abstract

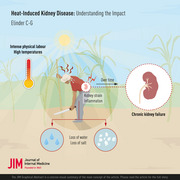

## Introduction

Some 20 years ago, health professionals in Central America became aware that a silent epidemic of chronic kidney disease (CKD) was affecting agricultural workers in Costa Rica, El Salvador, and Nicaragua [1]. The kidney disease was initially named Mesoamerican Nephropathy (MeN). Later, it has also been called chronic kidney disease of unknown cause (CKDu) or chronic kidney disease of nontraditional cause (CKDnt) in the absence of diabetes, hypertension, or any other specified kidney condition. The latter is the official name given by the Pan American Health Organisation (PAHO). Chronic Interstitial Nephritis in Agricultural Communities (CINAC) has also been suggested [2].

Initially, it was thought that this kidney disease, often affecting young male sugar cane cutters or agricultural workers, was an occupational disease caused by exposure to pesticides. Epidemiological studies, however, identified an association with heat stress rather than pesticides. At the first international workshop on MeN/CKDu in 2013, it was concluded that repeated episodes of dehydration with loss of electrolytes and minerals were the leading cause of the CKDu epidemic in Central America [3].

Parallel with the identification of an epidemic of CKD in Central America, reports came from Sri Lanka and India on a similar type of CKD that was affecting farmers in dry areas. In Sri Lanka, health professionals proposed that the Sri Lankan CKD epidemic was caused by toxic exposures of some type, such as heavy metals or pesticides, or from drinking water. The debate among researchers, epidemiologists, and toxicologists about the possible causes of CKDu/CKDnt has been going on since the beginning of 2010.

In this review, the evidence of the causes of what has been named MeN/CKDu/CKDnt has been scrutinized, using the principles that Hill presented in 1965 [4]. According to Hill, before deciding on mere association or causation, we should especially consider the observed associations regarding (1) strength, (2) consistency, (3) specificity, (4) temporality, (5) biological gradient or dose–response curve (6) plausibility, and (7) coherence. As a comparison, five of these factors (strength, consistency, specificity, temporality, and coherence) were used by the US Surgeon General's Advisory Committee in 1964 when agreeing that a causal relationship existed between lung cancer and smoking.

## Why is it important to conclude?

There is not yet a consensus among scientists on the cause(s) of MeN/CKDu/CKDnt. In recent reviews and reports, the potential causes have been mentioned, such as heat stress, heavy metals, pesticides, agrochemicals, and water quality, and further studies are needed. The following text is from the Executive Summary from the Fourth International Workshop on CKDu in Guatemala, February 2024: “There is no simple answer to what ‘causes’ CKDu. Important risk factors for kidney decline in populations at risk of CKDu have been identified; however, the etiology of the disease remains inadequately understood. Multiple factors are likely to contribute to CKDu, either independently, at different time points in the course of disease, perhaps together and/or in combination.” Table 1 presents a long list of potential exposures, other than heat stress, suggested to be causally related to MeN/CKDu/CKDnt.

**Table 1:** List of factors, other than heat‐strain, suggested as causative for MeN/CKDu/CKDnt.

Toxic agents Pesticides Agrochemicals Paraquat Glyphosate Aristolochic acid Ochratoxins Metabolic agents/disturbances Uric acid Fructose metabolism Metals/metalloids Cadmium Lead Mercury Arsenic Nickel Tungsten Lithium Silica Aluminum Metal corrosion Metal mixtures Infections Leptospirosis Hantavirus Other viruses Other bacterial infections Other parasitic infections Lifestyle/socioeconomic conditions Poverty, poor socioeconomic conditions “Environmental injustice” (regular NSAIDs use) Genetic predisposition Homemade alcohol, alcohol abuse
Hardness of water, water conductivity Water quality, surface vs. deep well/groundwater Miscellaneous Microplastics Mixtures of pollutants in various exposure media Inhalation of particles containing silica Other air pollutants Inhalation of particles, in work as well as at home

The ongoing debate is welcome as it makes it possible for researchers to continue searching for causes and for scientific discussion to continue. However, on the negative side, this lack of consensus slows down reasonable and important actions for prevention. Legislators and authorities are less willing to act if researchers and health professionals do not agree. Even worse, they may make wrong decisions. A government in Sri Lanka, which was eager to do something about the kidney epidemic, imposed a ban on the use of glyphosate, a much‐used herbicide [5]. This decision was based on a suggestion, but not on evidence, that glyphosate was the culprit of the CKDu epidemic in Sri Lanka. It caused serious problems for farmers, and the ban soon had to be lifted as important tea production in Sri Lanka was threatened.

Furthermore, time, effort, and research resources are spent on meaningless studies on agents that have repeatedly been shown not to cause MeN/CKDu/CKDnt, such as heavy metals. Meanwhile, it has been realized that MeN/CKDu/CKDnt, or a similar type of CKD, occurs in many parts of the world. Heat exposure and living in a hot climate have been associated with lowered renal function and increased incidence of CKD in many areas and countries worldwide where high ambient temperatures occur and where agricultural workers, construction workers, and other groups are exposed to heat stress. Susceptible groups in the general population do appear to suffer from acute kidney injury (AKI) and CKD more often if they live in hot e environments. We have a plausible and preventable cause, and it is time to act.

One may make a parallel to how long time and effort it took to reach a universal and unequivocal consensus that cigarette smoking was causing lung cancer and that anthropogenic carbon dioxide emissions are the most important cause of global warming today. Certain stakeholders, the tobacco and fossil fuel industries, respectively, did what they could to add uncertainty about the causal relationships and thereby prevented political and legislative actions for several decades [6]. This, in turn, caused thousands of cancer deaths [7] and a lack of effective actions to prevent further global warming [8].

It is important to distinguish among heat (environmental heat), heat stress (the combination of external heat, metabolic heat generated by muscular activity, and reduced heat dissipation from clothing, which results in increased core body temperature), and heat strain (the physiological changes caused by the increased core body temperature). Heat stress is the combined effect of external conditions, metabolic heat production, and clothing. Ambient temperature and humidity determine heat exposure, but other external factors can also have a large impact; convection will be reduced if there is no wind, evaporation is less effective the more humid the surrounding air is, and radiation is higher under full sunlight than shade. The Wet Bulb Globe Temperature (WBGT) incorporates environmental temperature, humidity, wind speed, and solar radiation and is commonly used for measuring heat exposure. A body at rest produces much less heat energy than one conducting physically heavy work. Clothing is an important determinant of heat energy transfers as clothes trap air heated and humidified by the body, limiting convection and evaporation [9].

A summary report on MeN/CKDu/CKDnt, where 418 scientific papers are cited and commented on, is available [10]. This summary report attempts to include everything that has been published in English on MeN/CKDu/CKDnt. Based on this report, we can now say for sure that heat stress is the driving force behind MeN/CKDu/CKDnt. The “u” in CKDu and the “nt” in CKDnt wrongly give the impression that we do not know its cause. A more appropriate name for the disease would, therefore, be heat‐induced kidney disease. This type of CKD may be severe but is preventable. If nothing is done, the prevalence worldwide of heat‐induced kidney disease is likely to increase further from continued global warming. The evidence is convincing, and the time for action has come. This includes making the public, politicians, and legislators aware of the threat to human health in a world that is rapidly getting warmer.

## History of recognition MeN/CKDu/CKDnt/heat‐induced kidney disease

At the beginning of the 21st century, the first reports on a CKD epidemic in Central America were published [11]. A new type of CKD was described as “not associated with diabetes, hypertension, primary glomerular diseases, or obstructive uropathy” affecting agricultural workers. A series of cross‐sectional studies revealed a high prevalence of lowered renal function with an estimated glomerular filtration rate (eGFR) below 60 mL/min per 1.73 m^2^, a proxy for CKD in epidemiological studies, in villages of Northwestern Nicaragua [12]. In a survey of 1096 persons in five villages, mining/subsistence farming, banana/sugarcane, fishing, service, and coffee village types took part in a health examination. In the mining/subsidence farming and banana/sugarcane villages, the prevalence of CKD was high among men, 18% and 17%, respectively. It was intermediate (10%) in the fishing village and lower in the service (0%) and coffee village (7%). The pattern was similar for women but with a lower prevalence. Proteinuria, measured by paper strips, was recorded in about one third of those with CKD. It was noted that the villages with a high CKD prevalence were located close to the coast and at a low altitude. It was suggested that a heavy workload in a hot climate leading to repeated dehydration may be an explanation. Another two studies [13, 14] reported a high prevalence (10%–18%) of CKD among relatively young men engaged in farming and agricultural work and exposed to heat during heavy workload, for example, sugarcane cutting. Only a few of these had proteinuria or hypertension.

An editorial in the American Journal of Kidney Disease called attention to the epidemic of CKD in Central America and mentioned that it “results in many thousands of deaths” by referring to national statistics in Nicaragua and El Salvador [15]. A similar type of CKD has also been reported by farmers in certain areas of Sri Lanka [16, 17]. In Sri Lanka, the prevalence of CKD was lower and usually seen at a higher age than in Central America. Screening programs for diagnosing CKD in Sri Lanka often included the identification of albuminuria as a first step.

The incidence and mortality of CKD were reported to be much higher in MeN/CKDu endemic areas along the Pacific coast in Costa Rica, El Salvador, Guatemala, and Nicaragua compared to other non‐endemic areas and regions in Central America [18, 19].

Perhaps the first report on heat‐induced kidney disease was from South Africa in 1970. Acute renal damage was seen as a common and important complication after heatstroke among gold miners, often under severe heat stress. Four out of 40 gold miners who underwent renal biopsy developed chronic progressive interstitial nephritis with persistent or progressive impairment of renal function (CKD) [20].

## Diagnosis of chronic and acute MeN/CKDu/CKDnt/heat‐induced kidney disease in Central America

MeN/CKDu patients in Central America typically are slim young or middle‐aged men working in the sugar plantation, presenting with elevated *p*‐creatinine, no or limited proteinuria, blunt urinary sediment, and no hypertension. This is not a typical CKD patient in the United States, Europe, or Japan. This explains the often‐used diagnostic term CKDnt (where “nt” stands for nontraditional). The first detailed clinical and renal pathological characterization of patients with MeN was presented in 2013 [21]. Eight cases from El Salvador showed signs of chronic glomerular ischemia in combination with tubular atrophy and interstitial fibrosis but only mild vascular lesions. Very similar findings were later seen in renal biopsies from patients with MeN in Nicaragua [22]. Individuals with MeN/CKDu/CKDnt often display hyponatremia, hypokalemia, and hypomagnesemia [23, 24]. Patients with CKDu from Sri Lanka [25] and India [26] have also been examined. They are often older than those from Mesoamerica, have no hypertension or marked proteinuria, and the renal pathology is similar but shows more interstitial inflammation.

Even if CKD is a chronic disease, AKI may occur during severe heat exposure. In a large group of agricultural workers (about 15,000), 1.6% of all, mostly young (median 29 years) males, had acutely elevated creatinine indicative of AKI. Almost all complained of nausea (59%), back pain, fever, vomiting, headache, and muscle weakness. Blood tests revealed leucocytosis and neutrophilia. In the urine, almost all patients displayed leukocyturia, hematuria was also common (82%), and 34% had albuminuria exceeding 0.3 g/L. Bacteriuria was not seen. Renal biopsies from 11 patients with suspected AKI from MeN showed tubulointerstitial nephritis with varying degrees of inflammation and chronicity [27]. This suggests that the chronic glomerular changes seen in typical cases of MeN may be preceded by acute interstitial inflammation in the kidneys. Sugarcane workers in Nicaragua and Guatemala who experience AKI more often develop CKD [28, 29].

One group of investigators claims that renal biopsy findings from patients with MeN/CKDu/CKDnt are typical of renal toxicity from “agrochemicals” with dysmorphic lysosomes and electron‐dense aggregates in renal tubular cells, and a new acronym was suggested: CINAC [2, 30]. However, this has not been supported by others [31], as similar types of dysmorphic lysosomes in proximal tubular cells are seen in renal biopsies from patients with other renal diagnoses as well [32].

## Prevalence and incidence MeN/CKDu/CKDnt/heat‐caused Kidney in “Hot‐spots”

The prevalence and incidence of CKD in certain seriously affected populations may be alarmingly high. In a community near the town of Chichigalpa in Nicaragua, sometimes named the “village of widows” as so many men die from MeN/CKDu/CKDnt, the prevalence of CKD was 42% among men and 10% in women. Hypertension was more prevalent among cases than controls, although the overall prevalence of hypertension was low, 8.6%. Aside from age and male sex, the strongest independent association observed was between reduced glomerular filtration rate (GFR) and lifetime hours cutting sugarcane, particularly during the dry season [33].

Repeated measurements of eGFR revealed that CKD occurred in men with an incidence rate of 0.7% per year in 2 community‐based cohorts (adults aged 18–30 years, *n* = 351 and 420) from 11 rural communities in Nicaragua followed up over 7 years [34]. This close to 1% per year incidence of CKD among men is at least 25 times that seen in the United States or Europe in similar age groups, confirming a huge burden of kidney disease in this population. Cumulative time in sugarcane work and symptoms of excess occupational sun exposure were associated with the development of CKD.

A high prevalence of CKD has also been reported in agricultural villages in Andhra Pradesh, India [35]. A total of 1201 subjects with a mean age of 45 were examined. The overall prevalence of CKD was 32%.

## Evidence that heat stress is the main cause of MeN/CKDu/CKDnt/heat‐induced kidney disease

Heat stress exposure is related to MeN/CKDu/CKDnt/heat‐induced kidney disease. It is a risk factor that displays a dose‐effect and dose–response relationship between intensity and duration of heat exposure on the one hand and prevalence of CKD, or severity, on the other [36]. Lifetime hours cutting sugarcane during the dry season is a major risk factor for CKD [33]. Renal tests over a 6‐month sugarcane harvest season in Nicaraguan sugarcane workers showed that those exposed to heat stress lost more of their renal function (eGFR) compared to workers not exposed to heat stress [37].

Environmental heat and workload are severe during sugarcane cutting [38]. Sugarcane cutters typically work on average for 7.5 h outdoors, with a WBGTs around 32°C. In a group of male sugarcane cutters in Nicaragua examined at the start of the harvest and at the end 5 months later, the pre‐shift renal function (eGFR) decreased significantly during 9 weeks of work in the cane cutters (9%), and mean urinary neutrophil gelatinase‐associated lipocalin (NGAL) increased indicating renal tubular stress [39].

Factors associated with a loss of kidney function (GFR) were examined in 520 young adults in a longitudinal study for 2 years. Agricultural work and lack of shade availability during work breaks were associated with a more rapid decline [40]. Of these workers, 81% remained stable, 9.5% experienced a rapid decline (−18.2 mL/min per drop in eGFR per year), and the remaining 9.5% had a lowered eGFR at baseline, had a decrease of eGFR of −3.8 mL/min per year.

The relationship between workload and the incidence of kidney injury was examined in a group of workers with different levels of physically demanding work [41]. The results provided evidence of dose‐effect as well as dose–response relations between high heat and high workload exposure and kidney injury. Data were collected before and at the end of harvest from four different groups: field support staff (low workload), drip irrigation workers (moderate), seed cutters (high), and cutters of burned sugarcane (very high). Mean cross‐harvest eGFR change was significantly associated with workload, increasing from 0 mL/min in the low‐moderate category to −5 mL/min in the high and −9 mL/min in the very high workload group. A similar pattern occurred for incident kidney injury (IKI), where low‐moderate workload had 2% compared with 27% in the very high workload category. Fever and C‐reactive protein elevation were associated with kidney injury.

A study on rice production workers is consistent with the concept that occupational heat exposure is a critical risk factor for MeN/CKDu/CKDnt in Mesoamerica [42] and not sugarcane cutting per se. Male field workers (*n* = 27) and other workers (*n* = 45) from a rice company were followed. Field workers were exposed above the recommended limits for occupational heat exposure during most of the shifts. Dehydration, defined as urine specific gravity ≥1.025, was common in both groups, but field workers were more exposed to heat and had higher workloads. Low eGFR was more prevalent in field workers at the start (19% vs. 4%) and follow‐up (26% vs. 7%), and field workers experienced incident kidney injury (IKI, s‐creatinine increase of ≥0.3 mg/dL over the study period, e.g., harvest) more frequently than other workers: 26% versus 2%, respectively.

Salt pan workers in India are also exposed to heat stress [43]. The mean WBGT was 30.5°C in summer and 27.8°C in winter. Water intake during the workday was low. Dehydration‐related symptoms were frequent in those with high‐heat stress, as were cross‐shift increases in body temperature >1°C (15%), a high urinary specific gravity >1.020 (28%), and a high sweat rate >1 L/h; (53%). The odds ratio for a lowered eGFR was higher in workers exceeding the threshold limit value for heat exposure. Exposures to pesticides and other toxicants are absent in the salt pans and allow the assessment of heat stress alone. Heat stress, dehydration, physical exertion, and lowered eGFR were also seen in 262 cashew workers in Tamil Nadu, India [44]. Occupational heat stress, as a possible risk factor for CKD, has not yet been investigated in Sri Lanka.

## Heat stress, markers, and the pathogenesis of heat‐induced kidney disease

Severe heat exposure during sugarcane harvesting has well been documented [45]. Sugarcane is often burnt the night before harvesting, and this adds to the ambient heat exposure. Already at 7:15, after a little more than 1 h of work, a preventive threshold of WBGT 26.0° was reached. After 4 h, 30.0° was passed, at which level no more than 15 min of work per hour is recommended to avoid acute health risks. However, the sugarcane cutters typically kept on for several more hours (until noon) to get their needed income, which is typically based on the amount (weight) of the cut. See Photos 1 and 2.

Experimental studies confirm that heat stress may influence renal function. Thirteen healthy adults (three females) exercised for 2 h at 40°C, 32% relative humidity in a four‐arm trial: water to remain well hydrated (Water), continuous upper body cooling (Cooling), a combination of both (Water + Cooling), or no intervention (Control) [46]. In the no‐intervention group, the core temperature increased by 1.9°C, and dehydration (percent loss of body mass) of 2.4 was the highest. There were greater increases in the urinary biomarkers of AKI in the no‐intervention group: Urine albumin and NGAL increased.

Pathophysiological mechanisms by which heat stress may induce kidney inflammation and, eventually, CKD in sugarcane workers have been presented [47]. In sugarcane cutters, inflammation biomarkers in plasma and fever are associated with AKI. Heat‐induced kidney injury could be mediated by inflammation caused by strenuous physical activity. Sugar consumption, pro‐inflammatory stimuli, including hypoxia, fructose, and uric acid, as well as circulating endotoxins and cytokines, may contribute. Sugarcane cutters often repeat the same activity for 6 or sometimes even 7 days a week for 5–6 consecutive months, meaning the possibilities for recuperative rest are limited.

Additional evidence has been obtained from experimental animals demonstrating that kidney injury can be mediated by a variety of factors, including body temperature, dehydration status, osmolality, concomitant sugar (fructose) ingestion, and the effects of vasopressin [48].

**Fig. 1:**
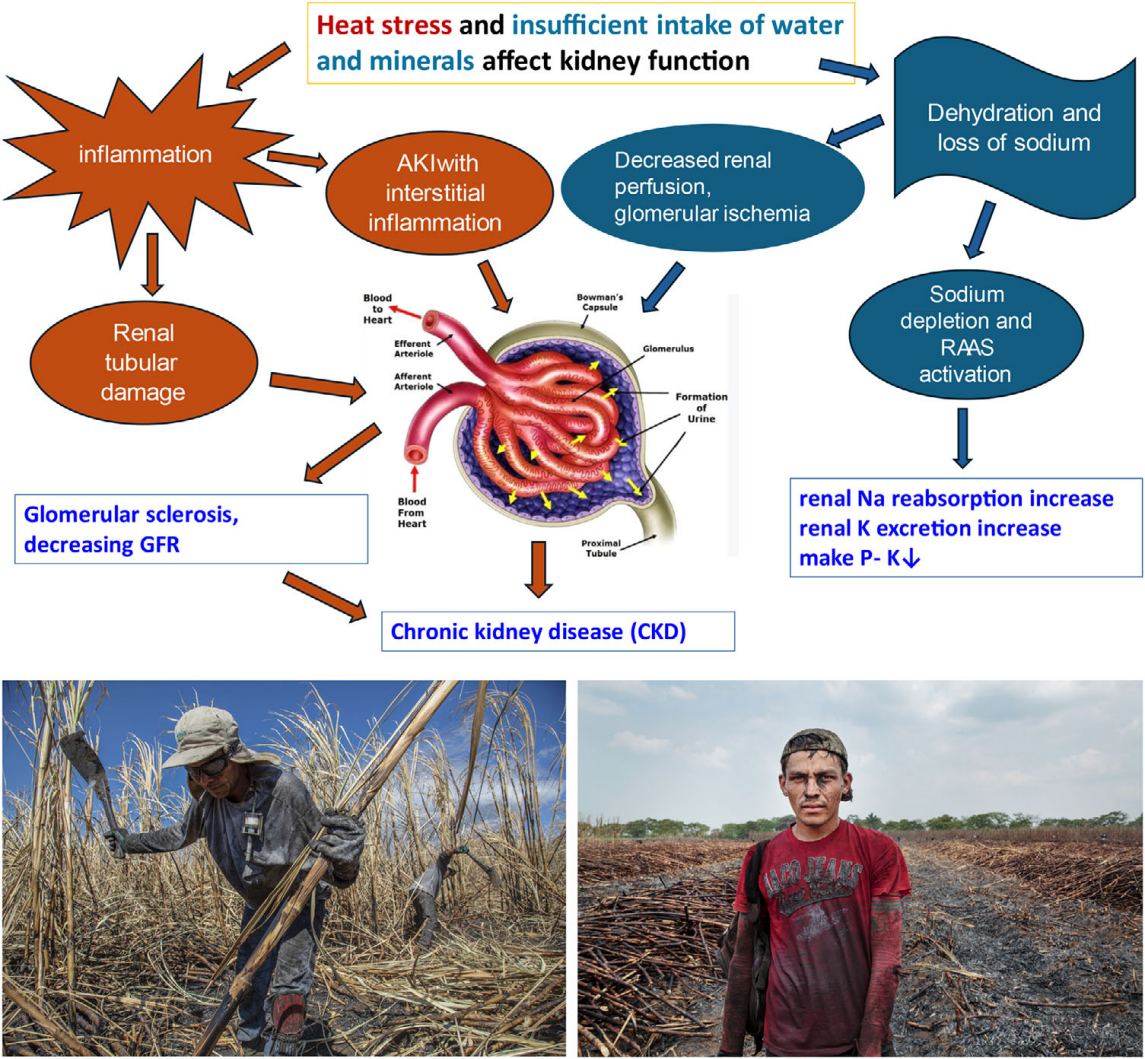
A schematic presentation of how heat stress and insufficient intake of water and minerals may cause chronic kidney disease (CKD). Photo 1: Sugar cane cutting in a recently burnt field Chichigalpa, Nicaragua 2020. Cutter carrying a monitor. Photo 2: Exhausted sugar cane cutter Chichigalpa, Nicaragua 2016. Source: Modified from Wernerson, 2012. Photo 1 and 2: Photographer Ed Kashi. With permission from La Isla Network [65].

It has also been suggested that low levels of sodium and/or potassium could have a role in the pathogenesis [49]. These changes may stimulate renal hormonal systems, for example, aldosterone, RAAS, and vasopressin, resulting in constriction of glomerular arterioles with glomerular ischemia and a mix of chronic glomerular and tubulointerstitial changes. See Fig. 1.

In a population at high risk of MeN/CKDu/CKDnt, 39 male Salvadoran sugarcane cutters were sampled on several occasions before and after work shifts during harvest [50]. Cutters worked at a high physical intensity at WBGT, mostly above 29°C for 6–8 h per day, 6 days a week, during the 5–6 months harvest season. Leukocyturia after work shift was the best marker of change in eGFR. Leukocyturia was associated with experiencing fever, little or dark urine, cramps, headache, dizziness, and abdominal pain. Decreasing blood hemoglobin and eGFR before harvest were also predictive of eGFR loss. Metabolomics, the study of patterns of small molecules derived from cellular metabolism and exogenous sources, is another approach to examine heat‐induced kidney disease [51]. Workers in high‐risk occupations were distinguishable by urinary metabolic features that suggest increased gut permeability, inflammation, and altered energy metabolism. Urinary quinoline‐to‐tryptophan ratio (Q/T) may also be a marker of ischemic and inflammatory AKI among men at risk for heat‐induced kidney disease [52]. In a sugarcane worker population of 693 45 developed AKI during the harvest season, urine Q/T was significantly higher in workers with AKI than in those with no kidney injury.

## A hot climate comprises an increased risk for CKD

Epidemiological data suggest that living in a hot climate increases the risk for AKI and CKD in the general population. The association between ambient temperature and hospitalization for renal diseases was examined in Brazil during 2000–2015 [53]. For every 1°C increase in daily mean temperature, the estimated risk of hospitalization for renal diseases increased by 0.9%. The associations between temperature and renal diseases remained for a few days. The risk was more prominent in females, children aged 0–4 years, and those aged over 79 years. However, neither MeN nor CKDu or CKDnT was mentioned in this report, which focused on acute hospitalizations rather than CKD.

A report in *The Lancet* [54] indicated that loss of renal function (GFR) is more rapid among patients with established CKD (having a urinary albumin‐to‐creatinine ratio of 200–5000 mg/g, and an eGFR of 25–75 mL/min) in areas with a hot climate. Climate and eGFR data were available for 4017 (93%) of 4304 participants participating in a randomized clinical trial in 21 countries. In adjusted linear mixed effect models, “heat index” was associated with a drop in eGFR. This effect was seen in the treatment as well as in the control group. This corresponds to an additional loss of 3.7 mL/min eGFR per year in a patient with an eGFR of 45 mL/min located in a very hot versus a temperate environment.

## Other exposures suggested to cause MeN/CKDu/CKDnt

The list is long, but the evidence is weak. It is well known that certain agrochemicals can cause serious health effects, particularly to farmers using toxic pesticides in countries and areas with insufficient and poorly applied regulations, and low knowledge of safe use. Reports of nephrotoxicity of pesticides, however, are rare [55, 56]. Paraquat, a pesticide with high acute toxicity, has often been suggested as a cause of MeN/CKDu/CKDnt [57]. However, the use of paraquat is very limited in sugar cane plantations, but it is much more used in banana plantations. Measured concentrations of pesticides in cane cutters have been low or undetectable [58]. MeN/CKDu/CKDnt in Central America is almost exclusively seen in the hot sugarcane plantation areas along the Pacific Coast of the Mesoamerica. Glyphosate has also often been suggested as a causative substance. It is a widely used herbicide all over the world, including in countries with both hot and more temperate climates. Glyphosate has a low acute toxicity, and the chronic human toxicity is likely to be low and still debated. In most of the epidemiological studies in Central America, different kinds of exposures have been examined, including heat stress and agrochemicals/pesticides. Here, dose–response associations have been seen between heat stress, or a proxy of that, on the one hand and lowered renal function (eGFR) on the other, but not for the use of pesticides or agrochemicals.

Toxic metals appeared early on the list in Sri Lanka. It is well known from the scientific literature that long‐term exposure to cadmium may cause kidney damage. However, the type of damage that cadmium causes in humans is not like that seen in CKDu. Exposure to cadmium in areas of Japan, where people have suffered from cadmium‐induced kidney disease, in drinking water and food has been many times higher than what has been recorded in Sri Lanka, or other countries with CKDu. The same applies to lead, arsenic, and other metals that have been proposed to cause MeN/CKDu/CKDnt [59].

Infections may cause kidney disease. However, none of the suggested infections in Table 1 has been associated with MeN/CKDu/CKDnt. Neither the clinical presentation nor the morphological findings in renal biopsies from MeN/CKDu/CKDnt patients resemble those of patients with these infections.

Lifestyle and socioeconomic conditions may be of importance. Many patients suffering from MeN/CKDu/CKDnt are poor, live in low‐income areas, and may have suboptimal nutrition and limited access to clean water. It is often informally employed manual workers that develop CKDu. Albeit dose–response relationships have not been reported, or typical renal pathology is seen, poverty and limited access to clean water likely contribute to the development of CKDu/CKDnt. Although some Sri Lankan reports suggest that the hardness of water, water conductivity, or surface versus deep well/ground water is causing CKDu, this has not been proven.

It has been suggested that exposure via inhalation to silica may have a role in developing MeN/CKDu/CKDnt. Sugarcane field workers in Guatemala are exposed to potentially high concentrations of particles containing amorphous silica or crystalline silica from the soil and from ash from the burning of cane before harvesting [60]. Measurements suggest that particles are occurring in size fractions that may impact the upper airways and penetrate the unciliated region of the lungs. Some support for the silica hypothesis has been presented in a research letter [61], which reports on the occurrence of amorphous silica nanoparticles (SiNPs) in human renal biopsies; six patients with typical MeN were compared with 16 control patients with focal segmental glomerulosclerosis (*n* = 6), membranous glomerulonephritis (*n* = 3), systemic lupus erythematosus (*n* = 3), and one each of IgA nephropathy, postinfectious GN, membranoproliferative GN, and thin basement membrane disease. The average number of amorphous SiNPs was higher in the MeN patients, but amorphous SiNPs were also found in the controls, and there was considerable overlap in the occurrence of amorphous SiNPs. If an arbitrary cutoff is set at 1 SiNP/100 µm^3^, all 6 cases with MeN showed an elevated number of particles, but also 6 out of 16 controls. Thus, the occurrence of silica particles is not diagnostic for MeN/CKDu/CKDnt and may well merely indicate that some type of silica exposure is more common in the area where cases come from than where control patients have resided [61].

## “Case closed” and the possibility of prevention

If all available data on the likely cause of MeN/CKDu/CKDnt is scrutinized and evaluated according to the established when assessing causality [4], it becomes obvious that heat stress is the likely culprit. Scientific evidence and facts are at hand, and it is time to close the case. Global warming will increase the number of people at risk for dangerous heat exposure and kidney disease [62] if nothing is done to stop it.

Heat‐induced kidney disease is seen and reported in different countries and regions, not only from heat‐stressed sugarcane cutters but also from other occupational groups. Associations are strong, consistent, and specific, occur after acute and chronic exposure, display dose‐effect and dose–response relationships, and are plausible and coherent.

In addition to what is discussed in the text and summarized in Table 2, implementing a program that provides water, rest, and shade for sugar cane workers is preventive. This program has reduced symptoms and signs of dehydration and kidney function damage in sugarcane workers in El Salvador and Nicaragua [41, 63, 64] with less loss of GFR during the harvest season and fewer events of AKI.

**Table 2:** Factors to Consider [4], when assessing causality between exposure and disease, here MeN/CKDu/CKDnt/heat‐induced kidney disease.

Heat stress			Other suggested exposures	
	Confirmed?	Comment	Confirmed?	Comment
Strength	✓	High risk of CKD for individuals under severe and repeated heat stress exposure	x	No evidence/data
Consistency	✓	Reported from different populations in several countries from many populations, and different occupations	x	No evidence/data
Specificity	✓	Typical clinical picture, biochemical findings, and renal pathology	x	No evidence/data
Temporality	✓	Heat stress exposure always preceded acute and acute and chronic kidney disease	✓	Suggested effects relate to previous exposure
Biological gradient, or dose–response curve	✓	The risk of attaining CKD increases with time and intensity of heat stress exposure	x	No evidence/data
Plausibility	✓	Likely underlying pathophysiological mechanism has been proposed	✓	The pathophysiological mechanism for how silica may cause CKD has been proposed
Coherence, findings does not seriously conflict with the generally known facts	✓	Heat stress exposure may cause acute kidney injury. Macro epidemiological studies repeatedly show associations between heat stress exposure and increased incidence of CKD or decreasing GFR	x	No evidence/data

*Note*: ✓ = Yeas, x = no.

Abbreviations: CKD, chronic kidney disease; GFR, glomerular filtration rate.

However, the conclusion that heat stress causes MeN/CKDu/CKDnt does not imply that all exposures and conditions listed in Table 1 have no significance. Clinical and renal morphological changes seen in MeN/CKDu/CKDnt may be associated with other exposures and diseases, and heat stress does not always cause MeN/CKDu/CKDnt. A parallel can be made to lung cancer. That smoking is a major cause of lung cancer does not mean that lung cancer is always caused by smoking, and neither does smoking always produce lung cancer.

Improving working conditions by providing hydration, rest, and shade to heat‐stress‐exposed workers is the most important measure to prevent heat‐induced kidney disease, especially in these times of global warming where millions of humans are likely to be exposed to hazardous heat stress.

## Conflict of interest statement

The author declares no conflicts of interest.

## Data Availability

Data sharing is not applicable to this article as no new data were created or analyzed in this study.
